# Food Sources of Key Nutrients, Meal and Dietary Patterns among Children Aged 4–13 Years in Ibadan, Nigeria: Findings from the 2019 Kids Nutrition and Health Study

**DOI:** 10.3390/nu14010200

**Published:** 2021-12-31

**Authors:** Rasaki A. Sanusi, Dantong Wang, Oluwaseun Ariyo, Toluwalope E. Eyinla, Marie Tassy, Alison L. Eldridge, Anuoluwapo Ogundero, Oluwatosin Leshi, Yvonne M. Lenighan, Shirley I. Ejoh, Elizabeth Aleru

**Affiliations:** 1 Department of Human Nutrition and Dietetics, University of Ibadan, Ibadan 20084, Nigeria; ariyoseun@gmail.com (O.A.); toluemma@ymail.com (T.E.E.); anuoluwaogundero@gmail.com (A.O.); ttlesh@gmail.com (O.L.); shirleyejoh@gmail.com (S.I.E.); elizabethadedipe16@gmail.com (E.A.); 2 Nestlé Institute of Health Sciences, Nestlé Research, Vers-chez-les-Blanc, 1000 Lausanne, Switzerland; Dantong.Wang@rdls.nestle.com (D.W.); Marie.Tassy@rd.nestle.com (M.T.); Alison.Eldridge@rdls.nestle.com (A.L.E.); yvonnelenighan@gmail.com (Y.M.L.)

**Keywords:** eating occasions, dietary patterns, food sources, nutrients, children 4–13 years, child nutrition, Nigeria

## Abstract

This study examined the contribution of food to nutrient intake, meal and dietary patterns among children aged 4–8 and 9–13 years in the city of Ibadan, Nigeria. Multi-pass 24-hour dietary recalls were used to assess intakes. Prudent and traditional Southwestern Nigerian dietary patterns were identified among children. The top foods and beverages were defined by frequency and amount consumed. Meal patterns were described by the eating occasions, while cluster analysis probed dietary patterns. About 88% of children had at least three meals including breakfast (95%), lunch (85%), dinner (92%) and midmorning meals (48%), while about 60% ate snacks at least once daily. Sources of energy and key nutrients were limited (yam, cassava, rice, maize, bread and beans/peas/legumes). The amount consumed per consumer of cassava products (192.2, 256.0 g), yam (169.7, 256.0 g), legumes (115.3, 150.7 g), corn/maize (160.4, 195.2), and rice (138.4, 182.3 g) were high, while beef (15.2, 17.9 g), eggs (50.6, 49.2 g), fish (27.5, 30.6 g), milk (24.2, 27.0 g) and nuts and seeds (18.2, 19.7 g) were low for children ages 4–8 and 9–13 years, respectively. In conclusion, while the frequency of meals suggests a healthy pattern, the top foods could not provide adequate nutrient (especially micronutrient) intake, which is key to the development of the target population.

## 1. Introduction

Food is necessary for maintaining a body’s basal metabolism, which processes energy and nutrients to sustain life, strengthen the immune system promote recovery and recuperation from illness and diseases and aid in growth and development, especially in children and adolescents [1]. Appropriate food intake is key for optimal nutrition; however, child and adolescent malnutrition has become an intractable problem in sub-Saharan Africa [2,3,4,5,6]. In Nigeria, for example, the high prevalence of malnutrition in children has been attributed to poor dietary practices including meal skipping, snacking and infrequent intake of fruit, vegetables, milk and milk products [7,8]. Malnutrition among school-aged children and adolescents contributes to poor learning outcomes, high morbidity and mortality, and impeded growth and development. Furthermore, early onset malnutrition has been shown to have a notable effect on neurological development and cognition [9].

Meal patterns are emerging as one of the explanatory variables in the epidemiology of nutrition-related diseases, especially among children and adolescents [10,11,12]. Meal patterns can be described by the frequency and spacing of eating occasions—the consumption of any meal or beverage, the amount, type of food and time when it is eaten [10]—as well as the skipping and timing of meals. Increased frequency of eating occasions and snacks consumption has been linked to a substantial increase in energy intake of children [11]. It has also been reported that evening snacks are more detrimental to healthy weight than those eaten during the day, thereby providing a simple strategy for obesity control [12]. In the United States, increased total dietary energy intake has been directly linked to eating occasions and portion sizes [13]. This suggests that the quality, frequency and quantity of food consumed are important considerations for energy and nutrient-intake studies.

While the prevalence of malnutrition and its consequences among children under five years of age in Nigeria has been consistently high for the past two decades [6], it is not clear to what degree malnutrition persists among school-aged children and whether the prevalence of malnutrition in 4–8-year-olds is similar or different to that of 9–13-year-olds. Moreover, Nigeria has few published dietary intake studies concerning school-aged children. A food consumption study among these age groups, therefore, provides a credible basis for the assessment of dietary intake patterns beyond the evaluation of adequate nutrient intake [14]. In our previous publication, micronutrient inadequacy (calcium, iron, folate and vitamin A) was a common problem in the sampled children and was particularly severe in the older children [14]. This, therefore, provides a justification for probing the sources of nutrients and their dietary contribution.

The purpose of this research is to examine the food sources of several key nutrients and evaluate the meal and dietary patterns in children 4–8 and 9–13 years of age in Ibadan, Nigeria. This report therefore provides answers to the following research questions: (i) What is the average number of eating occasions of children in these age categories? (ii) What proportion of children consume breakfast daily? (iii) What are the top foods they consume? (iv) Which foods are the main sources of energy and selected key nutrients? (v) What patterns are identifiable in the foods reported?

## 2. Materials and Methods

### 2.1. Study Design

The Ibadan Kids Nutrition and Health Study (I-KNHS) was an observational, cross-sectional survey of dietary intake, anthropometry, lifestyle parameters and feeding practices of children aged 4–13 years in the city of Ibadan, South-Western Nigeria. The sample size was determined using the Fisher’s formula [15] for cross-sectional descriptive studies.

#### 2.1.1. Sampling Methods

Data were collected from the five local government areas (LGAs) of the city of Ibadan (Ibadan North LGA, Ibadan North-East LGA, Ibadan North-West LGA, Ibadan South-East LGA and Ibadan South-West LGA). Each LGA was divided into wards and it was from these 59 wards that sampling frames were constructed using a stratified random sampling design. A total of 955 children aged 4–13 were selected across various social strata. All wards were first stratified into two groups based on population density, then a ratio of 1:3 between high and low population density groups was selected randomly as a proxy to reflect the socioeconomic status of residents. Systematic random sampling was used to select eligible households and simple random sampling to select one child per household from a constructed sample frame of eligible children. A child was considered eligible for inclusion if he or she had resided in the study areas for at least two consecutive years and the caregiver was available to provide necessary information on food intake and dietary practices.

Data were collected by trained interviewers in the child’s home between June and July 2019. All interviewers were university graduates of human nutrition and other biological sciences. Study materials were pilot-tested in a non-sampled area. Training and re-training of interviewers/data collectors was done before and after the pilot test respectively. A pilot study was done which sharpened the instruments for data collection and provided opportunity to choose the best of the interviewers. Four monitors supervised field activities during data collection. Geographic position system (GPS) was used to pinpoint the location of each household and local guides were available to view and report to their communities. The study protocol and all materials were reviewed and Ethical approval certificates were obtained from Ministry of Health, Oyo State of Nigeria (AD13/479/1149 dated 18 March 2019) and University of Ibadan/University College Hospital Ethics Committee (UI/UCH19/0107 dated 15 May 2019). Approval was also obtained from each of the five LGAs. In each community, leaders and representatives were involved in mobilizing and informing the public about the study and its purpose before the household listing and selection of the eligible children and their parents, guardians or caregivers.

#### 2.1.2. Participants

The main participants in this survey were school-aged children; the younger age-group (4–8) was assisted by their parent, guardian or caregiver, while the older age-group (9–14) was assisted only when necessary or when they were asked to confirm any information given by the child. Each parent, guardian, caregiver gave written and signed informed consent for the child to participate. Sociodemographic and socioeconomic characteristics of the participants in I-KNHS have been previously published [14].

#### 2.1.3. Dietary Intake Data Collection

The food intake of the children was assessed using a 24 h multi-pass dietary recall method [16]. Demographic data were collected with an electronic device using Open Data Kit (ODK) software, while paper and pencil were used for the 24 h dietary recall only. Interviews were conducted by trained nutritionists. This interview method asked and listed all the foods and beverages consumed by the respondent in the previous 24 h (midnight to midnight). Several steps or passes were used: (i) a quick list of all the foods and beverages consumed over the previous 24 h, including the time each food or beverage was consumed and the location of the respondent; (ii) a clear description of the food or beverage, including brand name and the preparation method of the food as consumed (e.g., raw, boiled, baked, fried; (iii) the quantity of food or beverage consumed in household measures (spoons or ladles), monetary cost if purchased from a vendor, or use of food models to describe the portion of each item consumed; and (iv) a review of the recorded items to ensure completeness. Questions were asked concerning feast or fast days, illness of the respondent or consumption of any nutritional supplements. A non-consecutive day repeat 24 h dietary intake assessment was carried out for 20% of the respondents to enable the estimation of usual nutrient intake using the method developed by National Cancer Institute as described previously [14]. In the present study, 24 h recall on the first day was used for meal and food intake analysis. Quality control measures applied in the post-data collection phase included (a) a training session on the use of the relevant data entry software; (b) a conference method of data entry that allowed for peer monitoring and close supervision by the I-KNHS team; and (c) daily random checks for accuracy of data entry.

### 2.2. Data Processing and Analysis

Prior to the data analysis, cleaning was carried out on the extracted data and 12 respondents were eliminated due to incomplete dietary data. The amount of individual foods and beverages reported in the 24 h dietary recall was converted to grams (food) or milliliters (beverages). The food, beverages and their amounts were then entered by research assistants into Food Processor nutrition analysis software (ESHA^®^ Research Inc., Salem, OR, USA). The Food Processor was expanded to include forty-two (42) foods specific to Nigeria using composition databases from the Nigeria Food Composition Table/Database [17], the West African Food Composition Table [18,19] and Food Data Central from the United States Department of Agriculture [20].

The Statistical Analysis System SAS^®^ (version 9.4; Cary, NC, USA) was used to analyze the consumption of foods, frequency of consumption, and the contribution of foods to key nutrients. Statistical comparisons were made between the younger (4–8) and older (9–13) children. Eating occasions were classified by time of eating into five categories: breakfast, midmorning meal, lunch, dinner and snacks. The first meal eaten by the respondent between 5 and 11:59 a.m. was classified as “breakfast” while any other meal eaten after the first meal before 12:00 p.m. (noon) was termed “midmorning meal”. The first meal eaten between 12 and 5:59 p.m. was termed “lunch” while the first meal consumed on or after 6 p.m. was classified as “dinner”. Snacks were categorized as food or beverages consumed in between meals. The top foods and beverages were identified based on frequency of consumption and amount consumed per consumer. The commonly consumed foods and beverages were extracted and their respective amount consumed per consumer was ranked. For the dietary pattern analysis, a total of 113 food codes were aggregated into 19 food groups representative of the local diet (Appendix A, Table A1). The groups were converted to percentages of total daily energy (%TE), to derive dietary patterns proportional to energy intake, and subsequently standardized as z scores. A two-step cluster analysis was initially applied to identify the number of dietary clusters in the cohort. The first step of this method involved the formation of pre-clusters, based on the distance criterion; the second step applied a standard hierarchical clustering algorithm to these pre-clusters. In this analysis, two dominant dietary patterns were identified using this approach. k-Means subsequently characterized these patterns by separating participants into non-overlapping groups derived from Euclidean distance. Socio-demographic characteristics were analyzed using the χ^2^ statistic for categorical variables and independent Student’s *t*-tests were applied to assess differences in continuous variables.

## 3. Results

### 3.1. Eating Occasions

Of the 943 children, about 95% had breakfast; 85% had lunch; 92% had dinner or supper; but only 48% had a midmorning meal (Table 1). Comparatively, the younger children (83.3%) consumed more snacks than the older ones (76.0%) and had more eating occasions. A statistically significant difference was observed when the meal frequency (*p* = 0.0308) was compared between age categories. Most children consumed three (54%) or four (34%) meals per day, whereas only 11% ate two meals and about 1% had a single meal daily (Table 1). Most of the respondents (80%) ate at least one snack per day.

### 3.2. Top Foods and Beverages Consumed by Children

The most frequently consumed food groups for both age groups were pepper stew and rice (Table 2). Pepper stew was consumed by more than 80% of children. Together, rice and its mixed-dish recipes were also dominant, with close to three-quarters of the children consuming rice and an additional 15–20% consuming rice mixed dishes. Other food groups consumed by more than half of the children included biscuits (63.3, 46.3%), beans, peas and legumes (54.8, 54.6%), cassava (51.9, 53.9%) and fish (52.1, 52.5%) for the 4–8 and 9–13 groups, respectively. Nutrient-dense foods like milk (28.7, 20.3%) and beef (21.4, 25.8%) were consumed by less than a third of all children 4–8 and 9–13, respectively. Vegetables were consumed as soup with starchy staples and were among the least consumed food group by children of either age group. The amount consumed by the respondents showed, on average, that cassava products (gari, fufu, amala lafun) were most frequently consumed and in the largest quantity (212.3 g/day). Plant-based sources of protein were more commonly consumed than those from animal sources.

The amount of notable micronutrient dense foods consumed was also observed to be low. The amount of milk consumed was only 25.2 g/day and fruit were not among the top 25 foods (Table 2). Another notable finding is the low consumption of nutritional beverages which are usually nutrient dense due to fortification. Specifically, they had among the lowest frequency (20%) and per person (17.35 g) consumption. Overall, similar trends in food consumption patterns were observed across the two age categories in this study (Table 2) with higher consumption among the older age group. Furthermore, while cassava products showed the highest in consumption among the younger children, yam and its products ranked the highest with older children. Most of the differences between the two age categories were not statistically significant. However, the consumption of a few foods, notably yam, maize and rice dishes differed significantly when statistically compared across the age categories.

### 3.3. Sources of Energy and Key Nutrients

The contribution of each food and beverage group to the daily energy and nutrient intake of the respondents is presented in Table 3. Foods belonging to the grain, root and tuber groups contributed most to the energy intake of the children with six grain-based foods ranked among the top 10. The contribution of protein rich foods to the intake of protein was low, notable among which were those of animal origin––eggs (6.4 g/day), fish (8.7 g/day), beef (5.4 g/day) and milk (3.7 g/day)—which ranked among the lowest contributors. Pastries contributed the most to daily fat intake of 17.4 g/day. The contribution of the top foods to iron intake was also observed to be low; the highest being cassava products (3.3 g/day) and the least being rice (<0.1 g/day). The contribution of milk to calcium intake was the highest (132.2 g/day). This was similar to vitamin A intake where the highest sources were milk (38.0 g/day) and eggs with its products (80.2 g/day). Folate was supplied mostly by wheat flour products (noodles and pastries), legumes, eggs and nuts.

### 3.4. Dietary Patterns

Cluster analysis identified two different dietary patterns in this 4–13 years old population (Table 4). Pattern 1 was defined by higher energy contributions from yeast breads, milk and yogurt, vegetables, beverages, spreads and oils (*p* < 0.001) and reflected a more prudent dietary pattern. Pattern 2 presented higher energy contributions from meat, poultry, seafood, nuts, seeds, soy, roots and tubers, soups and stew, mixed grain dishes, candies and sugars and starchy vegetables (*p* < 0.001), characterizing a traditional Southwestern Nigerian dietary pattern. There were no significant differences in demographic characteristics across the two dietary patterns (Appendix A, Table A2). Mean daily nutrient intakes across the two dietary patterns are presented in Table 5. The prudent pattern presented higher mean daily intakes for a number of nutrients, including energy, protein, carbohydrates, fat, B vitamins, vitamin D, vitamin E, calcium, phosphorus and sodium (*p* < 0.05). The mean daily intake of iron, potassium, selenium and zinc was significantly higher in Pattern 2 (*p* < 0.05).

## 4. Discussion

To the best of our knowledge, this is the first comprehensive study in Nigeria that describes the eating occasions, commonly consumed foods, meal pattern and contribution of such foods to the energy and key nutrients among children aged 4–13 years. The nutrient intake of this target group is presented in another report [14].

Meal patterns differed somewhat by age category, with a higher percentage of younger children consuming breakfast and midmorning meals than older children, who mainly consumed lunch and supper or dinner. Of the five eating occasions reported in this study, one finding was that majority had at least three meals daily and one snacking occasion. In addition, the comparatively higher number of the eating occasions among the younger respondents was important. This pattern was similarly observed in frequency of snacking, which particularly concurs with the literature as a prevalent dietary behaviour among children [7,11]. The implications of these observations may suggest better nutrient adequacy in the younger age group since the consumption of nutrient-dense foods was generally low, and it could be offset by higher eating frequency, which is also suggested by Bamidele Bello et al. in a study done with primary school students in Nigeria [21].

Another finding from our study was that carbohydrates (grains, roots and tubers, rice and rice dishes) contributed the most to the daily energy intake. In contrast, fats were limited and therefore contributed less to the energy intake. Low intake of fat-rich foods could be due to an underestimation of reporting ready-to-eat foods (or snacks) consumed outside the home, which may not have been witnessed by the caregiver or parent responding on behalf of the sampled child. A closer examination of the data showed that doughnuts, sweet rolls and pastries, which were mainly consumed as snacks, were the third-highest contributors to energy intake and the highest contributors of fat intake in both age categories. In Western countries, studies have reported associations between increased sweet snack consumption and rising obesity rates in children of this age group [12,22]. Thus, it is especially important to focus on the quality of snacks when promoting nutrient intake and encourage nutrient-dense foods like milk, fruit and vegetables.

To our knowledge this is the first time two dietary patterns with different nutritional profiles were identified using cluster analysis. Both dietary patterns are suboptimal in some nutrients. The “prudent dietary pattern” is characterized by a significantly higher intake of energy, protein, fiber, most vitamins and calcium than the “traditional South-western Nigerian dietary pattern”, but a lower intake of iron, magnesium and zinc. The findings suggest the significance of milk products in promoting adequate nutrition and the advantageous position of cereals and products over roots and tubers as key staples and major source of energy. Even though the first pattern seemed to provide more nutrients than the second, neither of the two brought an optimal dietary intake.

Moreover, despite earlier findings that protein intake was generally adequate in these Nigerian schoolchildren [14], our study demonstrated that few animal-based foods contributed to protein intake, especially in the “prudent dietary pattern”. There was widespread low consumption of animal-source food (including dairy products) compared to plant-based sources (legumes and grains). Consuming an adequate amount of animal-source food rich in protein is critical to the growth and development of children and an important source of micronutrients like iron and zinc. Notably, there was a poor contribution of iron, zinc and vitamin A from the current diet. This is indicative of a potential shortfall in micronutrient intake by the children. Consumption of such foods predisposes them to inadequate nutrient intake, especially when supplements and fortified foods are not commonly consumed [22].

When diets are based on starchy staples with suboptimal consumption from other groups, the risk of malnutrition is high. Fruit and vegetables for example are important sources of micronutrients and dietary fibre, but it is worth noting that fruit did not feature prominently among the top foods consumed by the children. Furthermore, the vegetable consumption by the children was mostly as vegetable soups which are consumed with starchy staples. In addition, the proportion of accompanying vegetables in soups is usually small compared to the starchy staple which may lead to a low intake of dietary fiber [14]. Our results showed that vegetables made a very poor contribution to the key micronutrients of interest, indicating that they were not consumed sufficiently. This was consistent with similar findings [23,24] of micronutrient intake among children. Inadequate consumption of fruit and vegetables is a risk factor for the development of certain chronic diseases later in life. Therefore, nutritional education and promotion of the consumption of nutrient-dense food is needed to improve the diet quality and nutritional state of school age children.

The strength of this study includes a robust sampling method, large sample size, and the used of an updated local food composition table [14]. To complement to our previous report which focused on nutrient intake and adequacy [14], we analyzed another aspect of the dietary intake of Nigerian children of this age group; i.e., food consumption and its contribution to nutrient intake. Another strength is that for the first time in Nigeria, we deployed a statistical procedure-cluster analysis to probe for dietary patterns in the sampled population. Of note, this study was conducted in the urban area of Ibadan and findings may not be representative of the whole of Nigeria or Oyo state, of which Ibadan is the capital. However, we expect that similar results would be obtained in other urban centers and cities in the country if these categories of children are targeted and a similar methodology were used. Another limitation is that the study was cross-sectional, indicating that the findings cannot be used to depict dietary patterns or behaviour over time. In addition, this study was conducted in June and July, meaning that the food sources availability may not be the same year-round.

## 5. Conclusions

The traditional South-western Nigerian diet as well as a prudent dietary pattern were observed among the children, but both age groups had a low intake of fruit, animal-source protein, milk products and fortified foods. Top foods consumed were from the starchy roots and tubers, cereals and legume groups. Breakfast consumption was common among the children and many of them ate about three meals daily with one or two snacks. Although the frequency of meals suggests a healthy pattern, the top foods consumed could not provide adequate nutrient intake. Nutrition education is needed to promote nutrient-dense foods. Other nutrition interventions such as food fortification of commonly consumed foods could be scaled up nationally. Policies such as school meals can be further optimized to provide nutritious foods to the school-aged children.

## Figures and Tables

**Table 1:** Eating occasions of respondents.

Characteristic	Total	4–8-Year-Olds	9–13-Year-Olds	χ^2^
	*n* = 943	*n* = 509	*n* = 434	
	Number (%)	Number (%)	Number (%)	
Gender				
Male	457 (48.5)	250 (54.7)	207 (45.3)	0.7102
Female	486 (51.5)	260 (53.7)	226 (46.5)	
Socio economic level				
Highest (A and B)	228 (24.2)	127 (24.9)	101 (23.3)	0.2974
Middle (C)	334 (35.4)	188 (36.9)	146 (33.6)	
Lowest (D and E)	381 (40.4)	194 (38.2)	187 (43.1)	
** Eating occasion				
Breakfast	896 (95.0)	492 (96.7)	404 (93.1)	0.0614
Midmorning meal	457 (48.5)	276 (54.2)	181 (41.7)	
Lunch	805 (85.4)	431 (84.7)	374 (86.2)	
Dinner	867 (91.9)	460 (90.4)	407 (93.8)	
Meal frequency per day				
1	11(1.2)	6 (1.2)	5 (1.2)	0.0308 *
2	102 (10.8)	50 (9.8)	52 (12.0)	
3	512 (54.3)	260 (51.1)	252 (58.1)	
4	318 (33.7)	193 (37.9)	125 (28.2)	
Snack frequency per day				
0	190 (20.1)	93 (18.3)	97 (22.4)	0.4838
1	354 (37.5)	186 (36.5)	168 (38.7)	
2	212 (22.5)	125 (24.6)	87 (20.0)	
3	119 (12.6)	66 (13.0)	53 (12.2)	
4	43 (4.6)	23 (4.5)	20 (4.6)	
5	17 (1.8)	11 (2.2)	6 (1.4)	
6	8 (0.8)	5 (1.0)	3 (0.7)	

* Significant difference at *p* < 0.05 between the two age groups. ** Frequency and percentage represent proportion of total within age group.

**Table 2:** Percent consuming and amounts consumed per day for top foods and beverages among school-aged children in Ibadan, Nigeria.

	All (4–13 Years)		4–8 Years (*n* = 509)		9–13 Years (*n* = 434)		
Foods	% Consuming	Per Consumer (g)	% Consuming	Per Consumer (g)	% Consuming	Per Consumer (g)	*p*-Value *
Pepper Stew	87.0	29.9	87.6	27.4	86.2	33.1	0.2035
Rice	75.5	157.2	77.8	138.4	72.8	182.3	0.9359
Biscuits, muffins, quick breads	55.5	26.2	63.3	25.5	46.3	27.3	0.001
Beans, Peas, Legumes	54.7	131.7	54.8	115.3	54.6	150.7	0.3007
Cassava and products	52.8	212.3	51.9	192.2	53.9	233.3	0.1491
Fish	52.3	28.9	52.1	27.5	52.5	30.6	0.2177
Leafy Vegetable Soup	49.7	56.4	51.9	52	47.7	61.9	0.7647
Yeast breads	44.5	133.2	46.4	118.3	42.4	151.3	0.7158
Eggs and omelets	32.6	50	34.4	50.6	30.4	49.2	0.4913
Doughnuts, Sweet rolls, Pastries	30.8	53.8	30.7	47.6	30.9	61.2	0
Pasta, noodles, cooked grains	29.4	96	30.5	89.4	28.1	103.9	0.4932
Yam and products	26.9	211.2	25.7	169.7	28.3	256	0.0014
Sugars and Honey	24.9	17.1	22.9	15.2	27.2	18.9	0.001
Milk, whole	24.8	25.2	28.7	24.2	20.3	27	0.8087
Beef, excludes ground	23.4	16.4	21.4	15.2	25.8	17.5	0.0143
Corn (Maize)	22.8	174.5	24.2	160.4	21.2	195.2	0.0056
Nutritional Beverages	19.9	17.4	24.4	17	14.8	17.9	0.7403
Rice mixed dishes	18.0	179.7	15.5	163.4	21	195.6	0.0545
Nuts and seeds	15.9	19	14.5	18.2	17.5	19.7	0.3773
Soft drinks	11.2	279.9	11.7	252.2	10.6	313.7	0.2958
Okra Soup	8.1	59.5	6.9	56.6	9.5	62.2	0.7632
Cowskin	7.1	28.8	6.7	27.9	7.6	29.7	0.6478
Plantain	6.9	51.9	7.3	53.9	6.5	49	0.0001
Melon soup	5.9	88.1	5.9	82.1	6	95	0.0158
Durum Wheat Flour	5.3	88.8	4.9	76.4	5.8	101.2	0.3283

* Student’s *t*-test to compare the mean intake between 4–8- and 9–13-year groups.

**Table 3:** Top food sources of energy and key nutrients (per consumer) among school-aged children in Ibadan, Nigeria.

	Contribution to Daily Nutrient Intake							
	Macronutrients			Minerals			Vitamins	
Food Group	Energy (Kcal)	Protein (g)	Fat (g)	Iron (mg)	Calcium (mg)	Zinc (mg)	Vitamin A (mcg)	Folate (mcg)
Yeast breads	361	12.7	3.6	1.3	34.6	1.2	0.0	0.0
Durum Wheat Flour	320	11.3	0.9	1.1	15.1	0.9	0.0	64.0
Cassava and products	282	1.4	0.4	3.3	95.9	4.1	0.0	0.8
Doughnuts, Sweet rolls, Pastries	274	4.0	17.4	1.8	10.7	0.6	0.6	41.1
Rice mixed dishes	260	4.7	4.8	1.6	25.2	1.9	0.2	0.1
Yam and products	223	1.4	1.1	3.8	18.4	3.7	0.5	3.8
Rice	204	3.7	0.3	0.1	4.7	0.7	0.0	3.1
Beans, Peas, Legumes	202	11.1	5.0	1.2	4.3	2.6	0.1	27.7
Melon soup	180	12.3	8.9	0.4	0.1	0.2	-	-
Plantain	179	1.1	7.7	0.4	3.6	0.2	27.2	13.8
Corn (Maize)	144	3.4	1.0	0.3	6.5	1.9	5.6	9.5
Pasta, noodles, cooked grains	142	5.4	0.9	1.2	6.4	0.5	2.0	67.0
Soft drinks	129	0.0	0.0	0.0	0.5	0.1	0.0	23.5
Biscuits, muffins, quick breads	124	2.6	3.2	-	-	-	-	-
Nuts and seeds	106	4.4	8.9	0.3	10.9	0.5	0.0	17.9
Eggs and omelets	81	6.4	5.7	0.7	26.0	0.6	80.2	22.8
Milk, whole	71	3.7	3.9	0.1	132.2	0.5	38.0	5.2
Sugars and Honey	66	0.0	0.0	0.0	0.2	0.0	0.0	0.0
Nutritional Beverages	66	1.8	1.1	2.4	135.0	2.1	-	12.1
Cowskin	65	13.5	0.3	1.2	17.6	2.0	-	-
Fish	44	8.7	0.8	0.3	1.5	0.1	5.8	0.6
Leafy Vegetable Soup	44	3.7	1.9	0.1	0.2	0.1	-	-
Beef, excludes ground	37	5.4	1.5	0.5	1.2	0.8	0.0	1.4
Pepper Stew	35	1.1	3.1	0.1	0.0	0.0	6.4	2.2
Okra Soup	15	2.2	0.3	0.0	0.0	0.0	-	-

- Represents missing values from the current food composition table.

**Table 4:** Percentage contribution of energy intake from food groups across two dietary patterns identified from Cluster analysis.

Food Groups	Prudent Pattern (*n* = 474)		Traditional Southwestern Nigerian Pattern (*n* = 481)		
	Mean	SD	Mean	SD	*p*-Value *
Pasta and rice	19.7	15.5	20.7	15.0	0.947
Quick breads	6.0	7.0	4.9	6.4	0.245
Yeast breads and tortillas	21.2	14.6	1.5	5.1	<0.001
Meat and poultry	1.0	2.4	1.5	2.9	<0.001
Seafood	1.7	2.6	2.8	3.3	<0.001
Eggs	2.5	4.0	2.2	4.1	0.743
Nuts, seeds and soy	0.7	3.1	1.8	4.1	<0.001
Milk and Yogurt	1.7	3.1	0.9	2.5	<0.001
Fruit and Juice	0.2	1.5	0.2	1.1	0.641
Roots and tubers	7.9	8.6	28.1	13.8	<0.001
Vegetables	13.4	14.9	7.4	10.7	<0.001
Starchy vegetables	2.3	5.5	3.0	7.1	0.001
Soups and stews	5.4	4.4	8.7	6.1	<0.001
Rice, pasta and other grain based mixed dishes	5.4	11.1	6.3	12.6	0.010
Desserts and sweet snacks	6.4	11.8	6.5	12.0	0.954
Chips, crackers and savories	0.4	1.9	0.4	2.0	0.942
Candies and sugars	1.0	2.2	1.7	4.2	<0.001
Beverages	3.0	4.7	1.2	3.3	<0.001
Spreads and oils	0.3	1.2	0.2	1.3	0.039

* As assessed by independent sample Student’s *t*-test.

**Table 5:** Mean daily nutrient intakes across the dietary patterns identified from cluster analysis.

	Prudent Pattern (*n* = 474)		Traditional South-Western Nigerian Pattern (*n* = 481)		
	Mean	SD	Mean	SD	* *p*-Value
Energy (kcal)	1592.8	578.0	1414.7	494.5	<0.001
Protein (g)	55.2	22.7	43.2	18.4	<0.001
Carbohydrates (g)	259.0	96.7	235.8	88.3	<0.001
Fibre (g)	9.4	6.0	7.0	5.1	<0.001
Sugars (g)	18.2	21.8	14.8	39.3	0.105
Total Fat (g)	34.6	19.0	31.0	17.0	0.002
SFA (g)	4.3	4.4	3.5	3.5	0.002
MUFA (g)	4.4	5.0	4.4	4.2	0.990
PUFA (g)	4.3	7.8	4.7	7.5	0.467
Vitamin A (RE)	63.4	106.6	56.6	181.2	0.486
Thiamin (mg)	0.6	0.4	0.4	0.3	<0.001
Riboflavin (mg)	0.6	0.5	0.4	0.4	<0.001
Niacin (mg)	10.3	7.4	9.0	7.0	0.005
Pantothenic Acid (mg)	1.8	1.5	1.4	1.1	<0.001
Vitamin B6 (mg)	0.7	0.5	0.6	0.4	<0.001
Folate (µg)	97.2	86.1	77.5	72.4	<0.001
Vitamin B12 (µg)	1.3	1.3	1.1	1.0	0.005
Biotin (mg)	15.2	22.4	12.5	17.0	0.032
Vitamin C (mg)	7.9	14.9	6.2	14.3	0.063
Vitamin D (µg)	1.3	1.4	1.1	1.0	0.004
Vitamin E (mg)	2.0	1.4	1.0	0.9	<0.001
Vitamin K (mg)	4.4	7.4	4.2	6.6	0.583
Calcium (mg)	210.5	195.3	186.8	135.3	0.030
Iron (mg)	7.8	4.0	8.6	4.2	0.002
Magnesium	123.1	60.8	126.4	49.0	0.356
Phosphorus (mg)	855.0	626.6	721.4	433.3	<0.001
Potassium (mg)	777.5	530.3	864.2	600.6	0.018
Selenium (mg)	41.2	38.6	46.3	37.6	0.040
Sodium (mg)	1086.0	669.7	807.9	624.2	<0.001
Zinc (mg)	9.3	5.9	10.8	6.1	<0.001

* As assessed by independent samples *t*-test.

## Data Availability

The data presented in this study are available on request from the corresponding author. The dataset is not publicly available due to ethical reasons.

## Appendix A

**Table A1:** Table of food categories and food groups used for the study of school-aged children in Ibadan, Nigeria.

1. CEREALS/GRAINS	2. SOUPS AND STEWS	6. PROTEIN FOODS	7. STARCHY ROOTS AND TUBERS
a. Durum Wheat flour (Semo)	a. Vegetable soup	a. Beef	a. Cassava
-Semolina (unenriched)	-Bean soup (Gbegiri)	-Boiled meat	-Gari (Dry)
b. Grits and other cooked cereals	-Dikanut soup (Ogbonno)	-Fried meat	-Gari dough (Eba)
-Checkers custard	-Jute leaves soup (Ewedu)	-Roast beef	-*Amala* lafun
c. Ready-to-eat cereals	-Mixed vegetable (Efo-riro)	-Liver	-Fufu
-Oatmeal	-Melon +Veg (Efo and Egusi)	-Ponmo (Boiled cowskin)	b. Plantain
-Corn flakes	-Okro soup	b. Chicken	-Fried plantain
-Golden morn	b. Stews	-Boiled Chicken	-Plantain chips
d. Pasta, noodles	-Palm oil stew	-Fried Chicken	c. Potatoes
-Boiled spaghetti	-Vegetable oil stew	-Boiled egg	-boiled sweet potato
-Noodles	3. SNACKS AND SWEETS	-Fried Egg	d. Yam
-Macaroni	a. Savoury snacks	c. Lamb, Goat, game and others	-Fried Yam
e. Rice	-Meat pie	-Boiled goat meat	-Amala dudu (Yam)
-Boiled rice	-Sausage roll	-Boiled Snail	-Boiled Yam
-Rice mixed dishes (jollof rice and fried rice)	b. Doughnut, Sweet rolls and Pastries	d. Fish	-Pounded Yam
f. Yeast Breads	-Chinchin (Minimie)	-Boiled Tuna	8. ALL BEVERAGES
-Bread	-Puff-Puff	-Fried Tuna	a. Beverages
g. Corn (Maize)	-Doughnut	-Boiled Catfish	-Tea
-Eko/Agidi (White)	c. Popcorn	e. Pork	-Coffee with milk and Sugar
-Ogi/Pap (white and yellow)	d. Sugar and honey	-Fried pork	- Plain water
-Tuwo Masara	e. Tortilla, Corn, other Chips	-Boiled pork	b. Carbonated soft Drinks
-Boiled corn	-Cheese balls	f. Nuts and Seeds	-Soda (all brands)
	a. Biscuits	-Roasted nuts	9. FRUIT AND 100% FRUIT JUICE
	-Biscuits (all brands)	-Boiled nuts	a. Fruit (Non-Juice)
	4. DAIRY	g. Soy Products	-Apple
	a. Milk	-Soy bean cake (tofu)	-Banana
	-Powdered milk	-Soy milk	-Citrus fruit (Tangerine and Orange)
	-Evaporated milk	h. Beans and Peas	-Water melon
	b. Nutritional Beverages	-Boiled beans	-Mango
	-Milo	-Beans Porridge	-Pinéale
	-Bournvita	-Akara (Vegetable oil and palm oil)	b. Juices
	-Milk and Chocolate	-Moin-moin (Vegetable oil and palm oil)	-Orange juice
	c. Yogurt		
	5. CONDIMENTS, GRAVIES, SPREADS, SALAD DRESSINGS		
	-Margarine (Blueband)		

**Table A2:** Participant characteristics across the two dietary patterns identified from Cluster analysis.

	Pattern 1 (%)		Pattern 2 (%)		* *p*-Value
Age Category					
4–8 years	57.0		50.5		0.089
9–13 years	43.0		49.3		
Gender (Male, Female)	51.7	48.1	44.7	55.1	0.083
Socioeconomic category					
AB (High)	25.3		22.5		0.412
C (Medium)	34.0		37.7		
DE (Low)	40.7		39.8		

* As assessed by χ^2^ test. Pattern 1 (*n* = 474), Pattern 2 (*n* = 481).
